# Supplementary material for: Role and mechanism of NCAPD3 in promoting malignant behaviors in gastric cancer
Source: Front Pharmacol. 2024 Apr 22;15:1341039. doi: 10.3389/fphar.2024.1341039 (PMC11070777; doi:10.3389/fphar.2024.1341039)
Supplement: Supplementary file 11 [file DataSheet2.ZIP › GSEA/immunologic/my_analysis.Gsea.1599462003817/GSE369_PRE_VS_POST_IL6_INJECTION_SOCS3_KO_LIVER_UP.html]

Details for gene set GSE369\_PRE\_VS\_POST\_IL6\_INJECTION\_SOCS3\_KO\_LIVER\_UP[GSEA]

|  || Dataset | filtered\_dataset.sample\_info.cls#WT\_versus\_NCAPD3\_MUT |
| Phenotype | sample\_info.cls#WT\_versus\_NCAPD3\_MUT |
| Upregulated in class | NCAPD3\_MUT |
| GeneSet | GSE369\_PRE\_VS\_POST\_IL6\_INJECTION\_SOCS3\_KO\_LIVER\_UP |
| Enrichment Score (ES) | -0.48742142 |
| Normalized Enrichment Score (NES) | -2.66388 |
| Nominal p-value | 0.0 |
| FDR q-value | 0.0034723182 |
| FWER p-Value | 0.011 |
Table: GSEA Results Summary

  

Fig 1: Enrichment plot: GSE369\_PRE\_VS\_POST\_IL6\_INJECTION\_SOCS3\_KO\_LIVER\_UP      
 Profile of the Running ES Score & Positions of GeneSet Members on the Rank Ordered List

  

| SYMBOL | TITLE | RANK IN GENE LIST | RANK METRIC SCORE | RUNNING ES | CORE ENRICHMENT || 1 | 3303 | HSPA1A | 206 | 0.669 | -0.1092 | No |
| 2 | 960 | CD44 | 339 | 0.573 | -0.1706 | No |
| 3 | 84159 | ARID5B | 699 | 0.379 | -0.4083 | No |
| 4 | 2114 | ETS2 | 703 | 0.375 | -0.3879 | No |
| 5 | 6558 | SLC12A2 | 839 | 0.276 | -0.4693 | No |
| 6 | 8837 | CFLAR | 865 | -0.278 | -0.4707 | Yes |
| 7 | 6515 | SLC2A3 | 873 | -0.283 | -0.4588 | Yes |
| 8 | 571 | BACH1 | 884 | -0.300 | -0.4480 | Yes |
| 9 | 7453 | WARS | 900 | -0.325 | -0.4393 | Yes |
| 10 | 694 | BTG1 | 901 | -0.326 | -0.4197 | Yes |
| 11 | 10797 | MTHFD2 | 911 | -0.338 | -0.4059 | Yes |
| 12 | 1195 | CLK1 | 920 | -0.349 | -0.3907 | Yes |
| 13 | 8738 | CRADD | 922 | -0.349 | -0.3705 | Yes |
| 14 | 3475 | IFRD1 | 935 | -0.360 | -0.3575 | Yes |
| 15 | 8780 | RIOK3 | 948 | -0.367 | -0.3441 | Yes |
| 16 | 4864 | NPC1 | 964 | -0.384 | -0.3319 | Yes |
| 17 | 5621 | PRNP | 970 | -0.387 | -0.3123 | Yes |
| 18 | 9180 | OSMR | 981 | -0.394 | -0.2958 | Yes |
| 19 | 3516 | RBPJ | 1021 | -0.419 | -0.2989 | Yes |
| 20 | 23764 | MAFF | 1039 | -0.436 | -0.2850 | Yes |
| 21 | 81631 | MAP1LC3B | 1060 | -0.452 | -0.2724 | Yes |
| 22 | 1604 | CD55 | 1090 | -0.468 | -0.2653 | Yes |
| 23 | 11221 | DUSP10 | 1095 | -0.475 | -0.2396 | Yes |
| 24 | 8727 | CTNNAL1 | 1106 | -0.482 | -0.2179 | Yes |
| 25 | 2012 | EMP1 | 1119 | -0.494 | -0.1969 | Yes |
| 26 | 665 | BNIP3L | 1185 | -0.556 | -0.2107 | Yes |
| 27 | 27 | ABL2 | 1218 | -0.592 | -0.1982 | Yes |
| 28 | 1983 | EIF5 | 1219 | -0.595 | -0.1625 | Yes |
| 29 | 1649 | DDIT3 | 1341 | -0.779 | -0.2034 | Yes |
| 30 | 26136 | TES | 1361 | -0.833 | -0.1671 | Yes |
| 31 | 6782 | HSPA13 | 1394 | -1.064 | -0.1263 | Yes |
| 32 | 26959 | HBP1 | 1395 | -1.073 | -0.0617 | Yes |
| 33 | 2591 | GALNT3 | 1403 | -1.195 | 0.0051 | Yes |
Table: GSEA details [plain text format]

  

Fig 2: GSE369\_PRE\_VS\_POST\_IL6\_INJECTION\_SOCS3\_KO\_LIVER\_UP      
 Blue-Pink O' Gram in the Space of the Analyzed GeneSet

  

Fig 3: GSE369\_PRE\_VS\_POST\_IL6\_INJECTION\_SOCS3\_KO\_LIVER\_UP: Random ES distribution      
 Gene set null distribution of ES for **GSE369\_PRE\_VS\_POST\_IL6\_INJECTION\_SOCS3\_KO\_LIVER\_UP**
